# Early Bronchus-Associated Lymphoid Tissue Lymphoma Diagnosed with Immunoglobulin Heavy Chain Molecular Testing

**DOI:** 10.1155/2016/7056035

**Published:** 2016-03-31

**Authors:** Pen Li, Lawrence Cheung, Brian Chiu

**Affiliations:** ^1^Department of Medicine, University of Alberta, Edmonton, AB, Canada T6G 2G3; ^2^Division of Critical Care Medicine, University of Alberta, Edmonton, AB, Canada T6G 2G3; ^3^Department of Laboratory-Medicine and Pathology, University of Alberta, Edmonton, AB, Canada T6G 2B7

## Abstract

When extranodal marginal zone B-cell lymphoma of mucosa associated lymphoid tissue (MALT), a low grade B-cell lymphoma, arises in the lung it is referred to as bronchus-associated lymphoid tissue (BALT) lymphoma. We describe a patient with a history of Sjögren's syndrome and rheumatoid arthritis with dyspnea and imaging consistent with lymphoid interstitial pneumonia (LIP). However, while histology and immunohistochemistry lacked definitive features of a lymphoma, immunoglobulin heavy chain (IgH) polymerase chain reaction testing demonstrated B-cell monoclonality, consistent with an early BALT lymphoma.

## 1. Case Presentation

A 65-year-old female with a 10-year history of seropositive rheumatoid arthritis and secondary Sjögren's syndrome treated with infliximab monotherapy presented with 5 weeks of gradually increasing dyspnea, nonproductive cough, and 1-2 kg weight loss. She was given a one-week course of doxycycline without improvement. Eventually, her dyspnea worsened to the point that she was unable to walk one block. Apart from being a seven-pack-year ex-smoker, her medical history was unremarkable.

Physical exam revealed normal vital signs. She had mild crackles bilaterally on auscultation. Cardiac, abdominal, and musculoskeletal exams were unrevealing with no splenomegaly, lymphadenopathy, edema, or digital clubbing. Her blood work was unremarkable, including a normal complete blood count and differential, liver enzymes, lactate dehydrogenase (LDH), and creatinine.

**Figure 1:**
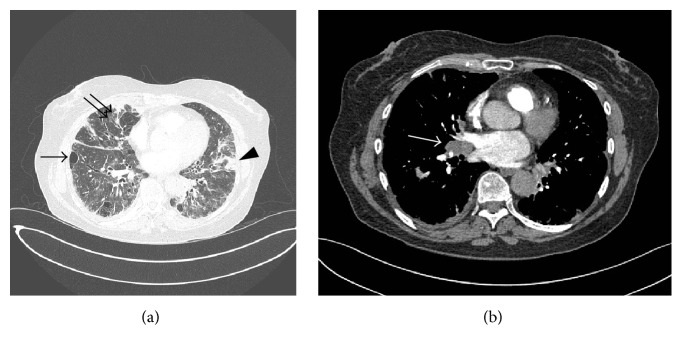
(a) Computed tomography scan of the chest, showing widespread pulmonary changes, including multiple thin-walled cystic air spaces (single arrow), scattered foci of consolidation (arrowhead), and interlobular septal thickening (double arrow). Radiologic findings were most suggestive of lymphoid interstitial pneumonia, but lymphoma could not be excluded. (b) Computed tomography scan of the chest demonstrating enlarged hilar nodes.

Chest X-ray revealed coarse reticular nodular markings in the mid to lower lung zones bilaterally with a predominantly peripheral distribution. These findings had slightly progressed compared to chest X-rays 4 weeks and 6 months before presentation but were not present 14 months before this presentation. A computed tomography (CT) scan of the chest (Figure 1(a)) demonstrated bilateral widespread abnormalities with basal predominance, including multiple thin-walled cystic air spaces, patchy ground-glass opacities, scattered foci of consolidation, some interlobular septal thickening, and a trace right pleural effusion. There were enlarged hilar lymph nodes up to 1.5 cm in size (Figure 1(b)). The imaging findings were most suggestive of LIP, especially in the context of Sjögren's syndrome and rheumatoid arthritis; however, since lymphoma could not be excluded and lymphadenopathy was not a usual feature of LIP, further investigations were done.

**Figure 2:**
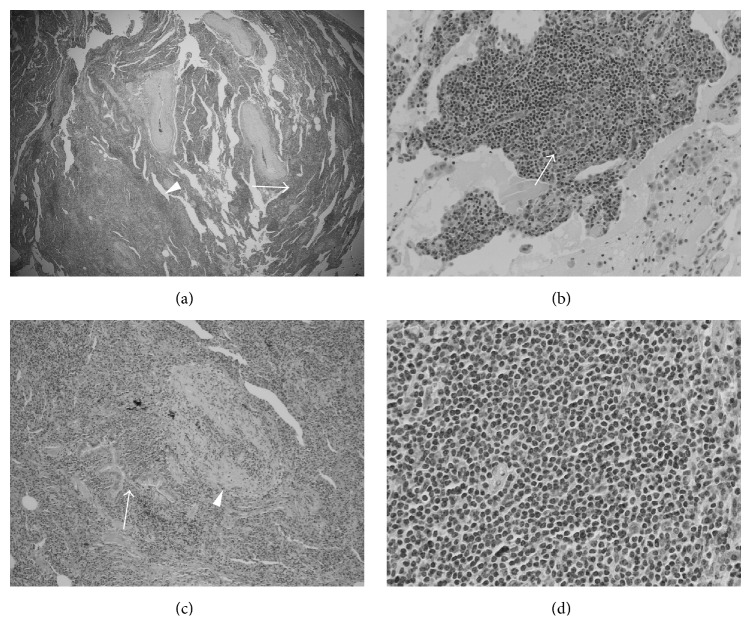
(a) Hematoxylin and eosin-stained lung wedge biopsy showing nodular and interstitial lymphoid infiltrates. Nodule (arrow head) interstitial infiltrate (arrow) (original magnification ×20). (b) Hematoxylin and eosin-stained lung wedge biopsy showing benign germinal centre (arrow) (original magnification ×1000). (c) Hematoxylin and eosin-stained lung wedge biopsy showing atypical lymphoplasmacytic infiltrate around bronchovascular bundle, around blood vessel (arrow head), and around bronchioles (arrow) (original magnification ×100). (d) Hematoxylin and eosin-stained lung wedge biopsy showing atypical small lymphoid cells within the nodular infiltrate (original magnification ×400).

Bronchoscopy with bronchoalveolar lavage of the right lower lobe and bronchial washing of the left lower lobe did not reveal microorganisms or malignant cells, and she did not respond to a trial of intravenous antibiotics. A video-assisted thoracoscopic lung biopsy of the right upper, middle, and lower lobes was performed 8 days after presentation to hospital. The biopsy revealed florid lymphoplasmacytic hyperplasia most suggestive of an LIP pattern related to her autoimmune diseases (Figures 2(a)–2(d)). There were no definitive features of a lymphoproliferative disorder; however, a few of the lymphoid follicles were somewhat nodular and obscured the lung architecture. As this was suspicious for lymphoma, subsequent B-cell receptor heavy chain (IgH) gene rearrangement assay testing was performed and demonstrated monoclonal B-cell proliferation, establishing the diagnosis of BALT lymphoma.

After confirmation of BALT lymphoma, she received 6 cycles of bendamustine and rituximab delivered on a 28-day cycle for 6 months. Her symptoms resolved and there was mild regression of the ground-glass opacities, foci of consolidation, and septal thickening on CT chest. She subsequently received rituximab every three months for maintenance therapy.

## 2. Discussion

Mucosa associated lymphoid tissue (MALT) lymphoma is a low grade B-cell lymphoma that arises in a number of glandular epithelial tissues including the stomach, lung, skin, bowel, conjunctiva, salivary glands, and thyroid [1]. MALT lymphoma involves the lung in about 15% of cases [2] and is referred to as BALT lymphoma.

Evidence suggests that the pathogenesis of MALT lymphomas relates to chronic antigenic stimulation, either by pathogens or by autoimmunity [1, 3]. Chronic inflammation is thought to perpetuate antigen-dependent B-cells, eventually leading to the proliferation of monoclonal B-cells. Initially, this early MALT lymphoma remains localized to the site of inflammation until further mutations occur to allow systemic spread.

BALT lymphoma is associated with connective tissue diseases including rheumatoid arthritis, systemic lupus erythematosus, and especially Sjögren's syndrome which increases the risk of developing lymphoma in general by 6.6–44 times [2]. Infectious associations with BALT lymphoma include* Mycobacterium avium* complex (MAC),* Mycobacterium tuberculosis* (TB), Epstein-Barr virus (EBV), and human herpesvirus 8; however, these associations are less established compared to other sites of MALT lymphoma, such as* Helicobacter pylori* in gastric MALT [3].

The most frequent reported symptoms in patients with BALT lymphoma include dry cough and dyspnea [2]. Patients may also, uncommonly, present with fever, night sweats, and weight loss. However, since many patients are asymptomatic or present with vague symptoms, there is often a delay in diagnosis. The largest review analyzed 63 cases of BALT lymphoma and reported that the median duration between initial symptoms and radiological abnormalities to diagnosis was 9 months, in which 44% of patients had presented with disseminated disease [2]. They found that laboratory findings were frequently normal, but some patients demonstrated anemia, thrombocytopenia, and elevated LDH levels. About one-third of patients produced a monoclonal gammopathy.

The most frequent patterns seen on CT scan of the chest include single or multiple nodules and areas of consolidation. Other potential findings include micronodules, ground-glass opacities, masses, septal lines, bronchiectasis, lymphadenopathy, and pleural effusions [2, 4]. ^18^F-2-Fluoro-2-deoxy-D-glucose- (FDG-) positron emission tomography (PET) scans often show mild uptake in the affected lung regions. False negatives are not uncommon, with reported sensitivities of 50–89%, given the indolent nature of this lymphoma [2].

Tissue biopsy is necessary for diagnosis and may be acquired by mucosal or transbronchial biopsies, CT-guided percutaneous needle biopsies, or surgical biopsies [2]. Pathological samples demonstrate polymorphous infiltrates of small lymphocytes, plasma cells, and B-cells. The B-cells infiltrate the epithelium and form characteristic lymphoepithelial lesions. About 14% of patients may have bone marrow involvement [2].

In this case, there were no definitive features of a lymphoproliferative disorder on histology and immunohistochemistry; however, a few of the areas of lymphoid hyperplasia appeared nodular and the suspicion of BALT lymphoma was raised. BALT lymphoma was confirmed when subsequent IgH molecular assay demonstrated monoclonality of the B-cell infiltrates and distinguished a low grade BALT lymphoma from a polyclonal LIP. This patient likely initially developed connective tissue disease-associated LIP which subsequently transformed into a lymphoma, which occurs in approximately 5% of cases [5].

The IgH gene encodes heavy chains for immunoglobulins and undergoes rearrangement early in the development of B-cells [6]. In a normal, polyclonal population of B-cells, each cell has a unique arrangement, resulting in a heterogeneous pattern when analyzed by polymerase chain reaction (PCR). In contrast, a monoclonal population of B-cells will produce a homogenous pattern. In the appropriate clinical context, the identification of a monoclonal IgH gene rearrangement confirms the diagnosis of a B-cell lymphoproliferative disorder. The sensitivity of this assay for nonfollicular B-cell lymphomas, such as BALT, is up to 84% and is about 63% for all B-cell lymphomas [6]. The specificity for B-cell lymphomas is about 94%, as some T-cell leukemias and lymphomas may produce a false positive result. The assay can be performed on peripheral blood, bone marrow aspirates, frozen tissue samples, or paraffin embedded tissue blocks.

The histologic differential diagnosis for LIP includes primary pulmonary lymphoma, hypersensitivity pneumonitis, and viral pneumonia (especially EBV) [5]. It is important to differentiate LIP from lymphoma, in our case BALT lymphoma, because of the different approach to therapy. Symptomatic patients with LIP who demonstrate progressive or severe disease are generally treated by immunosuppression with oral glucocorticoids or other medications such as azathioprine or cyclophosphamide. Conversely, current international treatment guidelines for BALT lymphoma [7] recommend first-line chemotherapy which typically includes an alkylating agent (such as chlorambucil, bendamustine, or cyclophosphamide) or purine analog (fludarabine), with or without rituximab. Surgery can be performed in patients with localized disease. Radiotherapy is reserved for patients with small lesions and/or contraindications to surgery. Overall, BALT lymphoma has a favorable prognosis with a 5-year survival of >85%, even with disseminated disease [2, 8].

This report describes the diagnosis of an early BALT lymphoma by demonstrating B-cell monoclonality with IgH molecular testing. We believe this test should be done whenever there are clinical, radiographic, or histologic suspicions for lymphoma but a lack of definitive histologic features on biopsy. Differentiating between an autoimmune or neoplastic process is important because of differences in therapy and prognosis. Further study is needed to determine the utility of routine IgH molecular testing on histologic specimens which show features of LIP alone but may, in fact, represent BALT lymphoma.

## Additional Points

*Learning Objectives*. (i) To recognize bronchus-associated lymphoid tissue (BALT) lymphoma as a pulmonary manifestation of connective tissue diseases, which may coexist with other lung pathologies. (ii) To understand immunoglobulin heavy chain (IgH) molecular testing and its clinical utility to diagnose early BALT lymphoma.

*CanMEDS Competency*. Medical Expert.

*Pretest Questions*. (i) What are the risk factors for developing BALT lymphoma? (ii) What is the histologic differential diagnosis for lymphoid interstitial pneumonia (LIP)?

*Pretest Answers*. (i) What are the risk factors for developing BALT lymphoma? BALT lymphoma is an antigen-driven lymphoma and is associated with chronic antigenic stimulation such as from autoimmune diseases, as well as chronic lung infections. (ii) What is the histologic differential diagnosis for LIP? The most important differentiation is between LIP and lymphoma. Other considerations include hypersensitivity pneumonitis and viral pneumonia.
